# Relationship between Mortality and Oral Function of Older People Requiring Long-Term Care in Rural Areas of Japan: A Four-Year Prospective Cohort Study

**DOI:** 10.3390/ijerph18041723

**Published:** 2021-02-10

**Authors:** Shiho Morishita, Yuki Ohara, Masanori Iwasaki, Ayako Edahiro, Keiko Motokawa, Maki Shirobe, Junichi Furuya, Yutaka Watanabe, Takeo Suga, Yayoi Kanehisa, Akitugu Ohuchi, Hirohiko Hirano

**Affiliations:** 1 Department of Oral Health Sciences, School of Health Sciences, Meikai University, 1 Akemi, Urayasu-City, Chiba 279-8550, Japan; s-morishita@meikai.ac.jp (S.M.); y-kanehisa@meikai.ac.jp (Y.K.); 2 Tokyo Metropolitan Institute of Gerontology, 35-2 Sakae-Cho, Itabashi-Ku, Tokyo 173-0015, Japan; iwasaki@tmig.or.jp (M.I.); aedahiro@tmig.or.jp (A.E.); kmoto@tmig.or.jp (K.M.); mshirobe@tmig.or.jp (M.S.); h-hiro@gd5.so-net.ne.jp (H.H.); 3 Division of Welfare, Faculty of Dentistry & Graduate School of Medical and Dental Sciences, Niigata University, 2-746 Asahimachi-Dori, Chuo-Ku, Niigata 951-8518, Japan; ohuchi@dent.niigata-u.ac.jp; 4 Department of Geriatric Dentistry, Showa University School of Dentistry, 2-1-1 Kitasenzoku, Ohta-Ku, Tokyo 145-8515, Japan; furuya-j@dent.showa-u.ac.jp; 5 Gerodontology, Department of Oral Health Science, Faculty of Dental Medicine, Hokkaido University, Nishi-7, Kita-13, Kita-ku, Sapporo 060-8586, Japan; ywata@den.hokudai.ac.jp; 6 Department of Geriatric Dentistry, Tsurumi University School of Dental Medicine, 2-1-3 Tsurumi, Yokohama 230-8501, Japan; suga-t@tsurumi-u.ac.jp; 7 Tokyo Metropolitan Geriatric Hospital, Tokyo 173-0015, Japan

**Keywords:** long-term care, longitudinal study, oral health, diabetes mellitus, mortality, geriatrics, oral dryness, survival analysis

## Abstract

Oral ingestion influences the life sustenance, quality of life, and dignity of older adults. Thus, it is an important issue in medical care and the welfare of older adults. The purpose of this four-year prospective cohort study was to investigate the relationship between mortality and oral function among older adults who required long-term care and were living in different settings in a rural area of Japan. This study included 289 participants aged 65 and older who required long-term care and lived in the former Omorimachi area in Yokote City, Akita Prefecture, located in northern Japan. Following the baseline survey, mortality data were collected over four years; 102 participants (35.3%) died during that time. A significant difference was noted in the overall survival rates between the groups with good and deterioration of oral function such as oral dryness, rinsing ability, swallowing function, and articulation, based on Log-rank test results. After adjusting for various potential confounders using Cox proportional-hazards regression, oral dryness (HR: 1.83, 95% confidence interval: 1.12−3.00) was significantly associated with mortality within four years. This study revealed that oral dryness influences the life prognosis of older adults who receive long-term care in different settings.

## 1. Introduction

As of 2020, older adults aged over 65 years account for 28.7% of Japan’s population—the highest proportion in the nation’s history and, currently, worldwide. The Japanese long-term care insurance system, which was established in 2000, provides social support for older adults and has become an indispensable part of their long-term care. According to the national registry, 2.18 million older adults were certified as requiring primary nursing care in 2000; however, by 2017, the number was 2.9 times larger, at 6.33 million [1].

Numerous reports are available regarding the importance of oral health among older adults [2,3,4,5,6,7]. Specifically, the maintenance of oral intake—which significantly affects the life prognosis, quality of life, and dignity of older adults—has become an important issue in medical care for and the welfare of older adults [8,9]. Decreased oral function, which is a contributing factor for oral intake difficulty, increases the risk of asphyxiation and aspiration pneumonia and leads to decreased appetite, calorie intake, and metabolism. This decrease in oral function can result in malnutrition and decreased physical function, affecting mortality [10].

In a study targeting independent community-dwelling older adults, Tanaka et al. [11] defined ‘oral frailty’ as multiple oral function decline. Those with oral frailty were found to be 2.35 times more likely to require long-term care and had 2.09 times higher mortality risk within four years than those without oral frailty. Even after adjusting for age, gender, activities of daily living (ADL), nutritional status, and cognitive function, the results indicated that deterioration of oral function affected frailty, sarcopenia, need for long-term care, and all-cause mortality. Considering these findings together, oral function may contribute to healthy life expectancy and biological life expectancy; however, how oral health status affects these health outcomes is still not completely understood.

Previous studies targeting older adults requiring admission to long-term care facilities reported that swallowing function and oral function relate to life prognosis after one year [12,13]. However, previous studies conducted in long-term care facilities have only sampled a few facilities of the same type or facilities in multiple communities. Older adults requiring long-term care are treated in different settings, such as day-care facilities, group homes for patients with dementia, rehabilitation facilities, intensive-care nursing homes, hospitals, and private residences. To validate the effects of oral function on life expectancy among older adults requiring long-term care, it is necessary to analyze longitudinal data with consideration to the diversity of their living environments.

Thus, we conducted this four-year prospective cohort study to investigate the relationship between mortality and oral function of older adults who require long-term care and are being treated in different settings in Japan’s rural areas.

## 2. Materials and Methods

### 2.1. Study Design and Participants

The participants of this study were older adults aged at least 65 years who required nursing care and lived in the former Omorimachi area in Yokote City, Akita Prefecture, located in northern Japan. This study began in 2013 and lasted four years. The participants received long-term care based on the Japanese long-term care system, as of February 2013, from day-care facilities and group homes for people with dementia. Additionally, some participants received care from two nursing homes, two wards in a general hospital, and home-visit nursing care. The study area included 2200 older adults, 470 of whom were certified as requiring long-term care by the public long-term care insurance system in Japan.

The Omorimachi area was selected because, unlike urban areas, only a small number of its older residents move from there to other communities. Further, there were few medical and long-term care facilities, which made it easier to conduct follow-up. The framework for the research in this area has already been outlined in previous studies [14,15].

**Figure 1:**
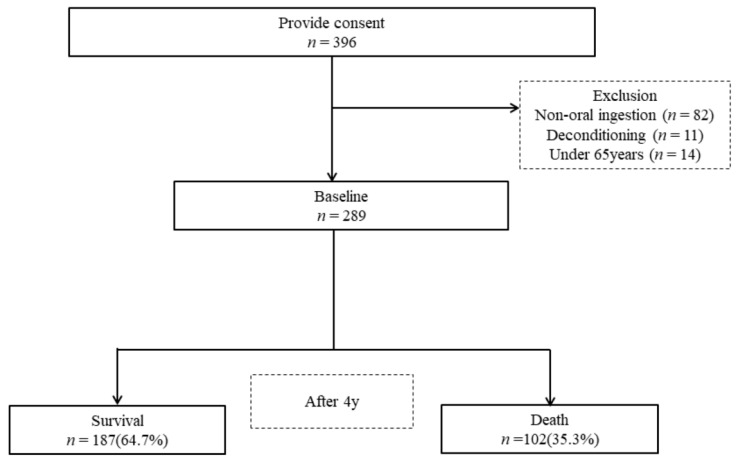
Flowchart of the participants in our study.

A baseline survey was conducted with 396 participants (67 men and 222 women) who consented to be included in the research. Figure 1 shows the participant flowchart for this study. As a result of the baseline survey, 107 individuals were excluded: 82 patients who received parenteral nutrition, 11 individuals who did not complete the survey because of symptom aggravation, and 14 individuals who were under 65 years of age. Thus, the primary study ultimately included 289 participants (mean age: 85.2 ± 7.2 years; 67 men, 222 women). Information regarding participant mortality was collected by sending a questionnaire to the facility or hospital four years later, in February 2017.

This research project was approved by the Ethics Review Board of the Research Department of the Metropolitan Geriatric Hospital and Institute of Gerontology (No. 38, 2009). The study’s purpose and details were explained to the participants or their guardians beforehand; only those who consented to the survey were included. All data were anonymized and managed under conditions designed to prevent identification of the participants.

### 2.2. Survey Items

In February 2013, we explained how the questionnaire should be completed to facility staff, nurses, and registered dietitians who provided care to the participants. The oral health status and function assessments were performed by dentists with at least two years of clinical experience who had participated in a two-hour training session. One of the dentists was certified by the Japanese Society of Gerontology. The training manual included an overview of the study and appropriate examination and data collection methods, and it had been constructed by the authors for the survey. The examiners were trained with volunteer participants until an agreement was reached regarding the criteria.

### 2.3. Basic Information

The participants’ age, gender, height, weight, medical history, and number of prescribed medications were transcribed from nursing care records.

### 2.4. Evaluation of Activities of Daily Living

The participants’ basic ADLs were evaluated using the Barthel Index (BI), which ranges from 0 to 100 points [16]. The BI is an ordinal scale to measure performance regarding ten variables describing mobility and ADLs: feeding, bathing, grooming, dressing, controlling bowels, controlling bladder, getting on and off a toilet, transfer from chair to bed, walking, and ascending and descending stairs. Higher scores reflect higher ADL function.

### 2.5. Evaluation of Cognitive Function

The facility staff used the Clinical Dementia Rating (CDR) to evaluate participants’ cognitive function [17]. The CDR determines the severity of dementia on a five-point scale (CDR = 0: healthy, 0.5: suspected dementia, 1: mild dementia, 2: moderate dementia, 3: severe dementia) [18]. In this study, ratings of 0 and 0.5 were combined into one category.

### 2.6. Nutritional Status

#### 2.6.1. Mini Nutritional Assessment^®^—Short Form (MNA^®^-SF)

Nutritional evaluations were conducted by a registered dietitian using the Mini Nutritional Assessment-Short Form (MNA^®^-SF) [19,20]. The MNA^®^-SF comprises six items: decrease in food intake over the past three months, weight loss over the past three months, mobility, presence of acute disease or psychological stress over the past three months, neuro-psychological problems such as dementia and depression, and body mass index (BMI). Total scores for the MNA^®^-SF range from 0 to 14 points, with higher scores indicative of a better nutritional state. According to the criteria, the highest score is 14 points, 12 points or higher is normal, 8 to 11 points is at risk for malnutrition, and 7 points or lower is malnourished.

#### 2.6.2. Body Mass Index

Participants’ BMI was calculated based on their height and weight.

#### 2.6.3. Food Morphology

Food morphology of participants’ staple diet was evaluated by facility staff, nurses, and registered dietitians in charge of the participants based on a five-point scale in order of hardness, with reference to National Dysphagia Diet (NDD) [21]: regular diet, soft rice, porridge, soft porridge, and blender porridge. Food type: regular was classified under regular diet, while soft rice, porridge, soft porridge, and blender porridge were all classified under defined formula diet.

### 2.7. Oral Assessment

#### 2.7.1. Number of Teeth Present

The number of teeth was considered the number of present teeth, excluding missing teeth replaced by prosthesis such as dentures, pontic teeth, and dental implants. Third molars involved in occlusion were included, while residual roots not involved in occlusion were excluded.

#### 2.7.2. Rinsing Ability

Referencing the method used by Sato et al. [22], participants were deemed ‘able’ to rinse if they could inflate their cheeks repeatedly while simultaneously moving their tongue quickly. They were deemed ‘unable’ to rinse if they could only slightly inflate their cheeks or were unable to close their lips, inflate their cheeks, or move their tongue.

#### 2.7.3. Articulation of /TA/

The participants were asked to pronounce the single syllable /TA/ or pronounce words that included this syllable. We evaluated whether the syllable could be pronounced audibly and clearly. If it was difficult for the participant to verbally respond to instructions, the characters of each syllable were shown, visual instructions were added, and pronunciation was instructed for evaluation. The participant was classified as having a ‘good’ evaluation if their pronunciation was clear. Otherwise, they were classified as having a ‘poor’ evaluation. Moreover, if a participant found it difficult to respond to instructions verbally, their daily conversations were observed.

#### 2.7.4. Modified Water Swallowing Test (MWST)

MWST was used with cervical auscultation to evaluate swallowing function [23,24]. Following the conventional method, 3 mL of cold water were injected into the floor of the mouth using a 5 mL syringe, and the participant was instructed to swallow. Subsequently, the sound before and after swallowing and changes in the respiratory sound were evaluated. If moist or foamy sounds were present in the pharyngeal swallowing sound or wheezing or gag reflex was present, the swallowing sound was classified as abnormal [23]. Based on the method of Sakai et al. [25], participants who could not complete the test, those who got an MWST score of 3 or lower, and those who had abnormal results in the cervical auscultation test were evaluated as ‘poor’. Those with an MWST score of 4 or higher were evaluated as ‘good’.

#### 2.7.5. Oral Hygiene Status

Oral hygiene status was assessed by plaque and tongue coating status by examiner inspection. Dental plaque was indicated as ‘none’ when there was no dental plaque between the teeth or supragingival area. It was indicated as ‘present if dental plaque was found between the teeth and supragingival area, or if dental plaque was noticeable in the supragingival area. The tongue coating was labelled as ‘none’ if the entire tongue was uniformly pink. The tongue coating was labelled as ‘present when a white, yellow, or brown coating was found on more than half of the tongue.

#### 2.7.6. Oral Dryness

To evaluate oral dryness, saliva on the back of the tongue was evaluated on a four-point scale based on clinical diagnostic classification proposed by Kakinoki et al. [26]. This classification of oral dryness is frequently used in clinical practice, as rated on the following scale: 0, non-dry (does not show condition 1−3); 1, saliva shows viscosity; 2, saliva shows tiny bubbles on tongue; 3, dry tongue without viscosity, little or no saliva shown [26]. If a participant scored 1 (mild) or higher, oral dry-ness was marked as ‘present’.

### 2.8. Statistical Analysis

The participants were divided into ‘surviving’ and ‘deceased’ groups, and the differences in their characteristics from baseline were examined. A χ^2^ test was conducted for categorical variables, and the Mann−Whitney U test was conducted for continuous variables, after checking normality using the Shapiro−Wilks test.

The variables related to oral function (rinsing ability, evaluation of /TA/ articulation, MWST score, state of dental plaque, state of tongue coating, and oral dryness) were examined for significant differences between the surviving and deceased groups. A Log-rank test was used to determine whether these variables affected the overall survival rate, and the overall survival curve was estimated using the Kaplan−Meier method.

Next, to identify factors associated with death within four years, we performed multivariate regression analysis using the Cox proportional-hazards model on the target variables from the start of observation to the occurrence of death. The variables related to oral function entered the model were those which showed significant differences between the surviving and deceased groups. Furthermore, age, gender, BI, medical history (aspiration pneumonia, cerebrovascular disorder, cardiovascular disease, neoplastic diseases, and diabetes mellitus), number of medications, CDR, and MNA^®^-SF score were entered into the model as independent variables. To eliminate the possibility of multicollinearity, a variable was deleted if a Spearman correlation coefficient of 0.8 or higher was noted between variables. IBM SPSS 25 (IBM, Tokyo, Japan) was used for all statistical analyses. In all cases, a statistically significant difference was set at *p* < 0.05.

## 3. Results

Among the 289 respondents in the baseline survey conducted in February 2013, 102 (35.3%) were confirmed to have died by February 2017. There were no untraceable individuals. The median (interquartile range [IQR]) of the follow-up period for the entire population was 1461 days (1137–1461; Table 1).

Table 1 shows the participants’ baseline characteristics. Between the surviving and deceased groups, significant differences were noted in age, gender, BI, CDR, BMI, MNA^®^-SF score, food type, rinsing ability, MWST score, /TA/ articulation, and oral dryness.

The deceased group was significantly older and had a higher proportion of men than the surviving group. Furthermore, the deceased group had a significantly lower BI (index indicating ADLs) and a higher proportion of people with severe dementia based on the CDR. Among the nutrition-related items, the deceased group had a significantly lower BMI and a higher proportion of malnourishment based on MNA^®^-SF score. Regarding food type, the deceased group had a significantly higher proportion of participants on a defined formula diet. For the oral evaluation items, the deceased group had poor rinsing ability, MWST scores, and /TA/ articulation, and had a significantly higher proportion of participants with oral dryness.

**Figure 2:**
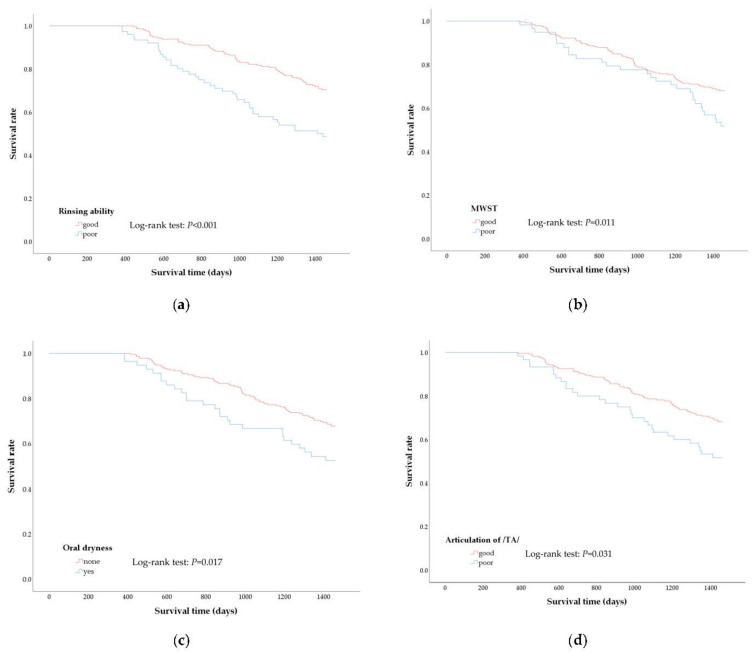
Kaplan–Meier curves and results of Log-rank test between poor and good oral function group. Kaplan-Meier curves represent the difference in survival rate according to oral function as assessed by rinsing ability (**a**), modified water swallowing test (**b**), oral dryness (**c**), and articulation of /Ta/ (**d**) during the 4-year observational period.

Figure 2 shows the survival curve produced using the Kaplan−Meier method for oral evaluation items (rinsing ability, MWST score, /TA/ articulation, and oral dryness); significant differences were observed between the surviving and deceased groups. Based on the Log-rank test results, significant differences were noted in the overall survival rates between the ‘good’ and ‘poor’ groups for all items. A significant difference was noted in the overall survival rate with oral dryness were 16.0% compared with 84.0% in group without oral dryness (*p* = 0.017). Similarly, significant differences were observed in the overall survival rate for rinsing ability, 19.8% in the poor group compared with 80.2% in the good group (*p* < 0.001), for articulation, 16.6% in the poor group compared with 83.4% in the good group (*p* = 0.031), and for MWST, 16.0% in the poor group compared with 84.0% in the good group (*p* = 0.011).

Table 2 shows the results of the multivariable Cox proportional-hazards regression analysis. Correlation analysis performed to eliminate multicollinearity prior to multivariate analysis failed to identify variables with strong correlations between independent variables. Age (HR: 1.07; 95% CI: 1.03−1.11), gender (woman; HR: 0.38; 95% CI: 0.23−0.63), BI (per one-point increase; HR: 0.98; 95% CI: 0.97−1.00), diabetes mellitus (HR: 1.80; 95% CI: 1.07−3.04), and oral dryness (HR: 1.83; 95% CI: 1.12−3.00) were statistically significantly associated with four-year mortality. The findings suggested that the presence of oral dryness, among other oral function variables, was likely to increase the risk of death by 1.83 times, even after controlling for various confounding factors.

The group with poor rinsing ability, pronunciation dexterity, and swallowing function, and presence of oral dryness had significantly shorter survivals compared with the ‘good’ group.

## 4. Discussion

We conducted this four-year prospective cohort study to investigate the relationship between mortality and oral function among older adults requiring long-term care in Japan’s rural areas. The results of a Cox proportional-hazards regression analysis revealed an association between oral dryness and the period of survival after four years.

To the best of our knowledge, this study is the first to clarify the relationship between oral dryness and mortality rates. Clinically, it is a significant finding that oral dryness was identified as a common risk factor for the occurrence of death in older adults requiring long-term care living in different settings in relation to factors such as lifestyle environment, even after adjusting for various confounding factors, such as ADLs and dementia severity.

Oral dryness frequently occurs among older adults and has been reported to be associated with disease, medication side effects, and dehydration [27,28,29,30]. This study’s findings did not show an association between number of medications and the occurrence of death; however, oral dryness is a symptom frequently noted in older adults who take multiple medications, experience dehydration, or have diabetes mellitus [31,32]. In addition, oral dryness also causes dysphagia [33,34] and induces a vicious cycle of conditions, such as undernourishment [35], decreased metabolism, susceptibility to infection, further dehydration, circulatory failure, and cardiac strain [36]. Thus, oral dryness may an important symptom that reflects overall health among older adults.

Furthermore, oral dryness leads to a decline in oral hygiene caused by decreases in the antibacterial and self-cleaning effects [37] of the oral cavity, and can also increase the risk of aspiration pneumonia [38,39]. Saliva performs various functions, such as cleaning, antibacterial protection, lubrication, masticatory assistance, mucosal protection, and aiding in digestion. It reflects the health of the oral cavity and plays an essential role in the normal functioning of the body.

Individuals with parenteral nutritional intake or who had difficulties completing the survey were not included in the analysis. As such, this could be deemed an incomplete survey. However, the results are based on approximately 60% of older adults requiring long-term care in the target community. If we included the 107 people excluded from the analysis, the survey would cover approximately 90% of the target community. Based on this, we believe that the study’s results generally reflect the characteristics of older adults requiring long-term care in Japan’s rural areas.

In the present study, MNA^®^-SF was not associated with mortality based on the results of the Cox proportional-hazards regression analysis. Hoshino et al. [13] reported similar results among nursing home residents. However, previous studies have reported an association between MNA^®^-SF and mortality [40,41,42]. We assumed that differences in the length of the observation period and the participants’ characteristics were responsible for the inconsistency of these results [40].

In this study, diabetes mellitus was associated with mortality, and these results were consistent with those of previous studies [43]. The diabetic group’s mortality was 1.76 times higher than all mortalities in the nondiabetic group and pneumonia mortality for the four-year period. Furthermore, the risk of mortality due to pneumonia was 3.25 times higher. This supports previous findings that aspiration pneumonia is the main cause of death in older adults [39]. In our analysis, the results showed that both diabetes mellitus and oral dryness were associated with mortality. Oral dryness was reported as a characteristic of patients with diabetes mellitus [44,45,46]. Oral ingestion is important for people with diabetes mellitus to maintain quality of life and control blood glucose levels. Since oral health management is essential, further studies are needed on its relevance to oral health for older adults requiring long-term care.

We performed a Log-rank test to examine the factors that differentiated oral assessment in terms of the survival period. As a result, a significant difference was observed between the ‘good’ and ‘poor’ groups for rinsing ability, MWST scores, /TA/ articulation, and oral dryness. These results suggest that decreases in oral function are related to survival time.

To perform rinsing requires coordination of oral, maxillofacial, and respiratory functions. Researchers reported that the inability to rinse was significantly associated with aspiration in older adults requiring long-term care [27,28]. Furthermore, poor articulation may be due to a systemic disease, deterioration of brain and nerve function, or deterioration of tongue movement [47] related to aging. Consequently, this may affect eating behavior and nutrition, and may lead to the deterioration of physical function and malnutrition [48]. Moreover, the deterioration of oral function is connected to aspiration and dysphagia, which increases the risk of aspiration pneumonia. Thus, there is sufficient evidence to support the fact that a significant difference was noted in the survival period depending on oral condition.

In previous studies targeting people with severe dementia in nursing homes, a decrease in MWST was associated with mortality within one year [13]. However, in this study, Cox proportional-hazards regression analysis revealed no significant difference. We assume that this discrepancy was because, in previous studies, oral dryness was not assessed and the observation period was different. Furthermore, this study targeted older adults requiring long-term care who were given nursing care in different settings, including home-bound patients. As such, it is possible that the range of ADL and cognitive function of all participants was wider than in previous studies.

In epidemiological studies among community-dwelling older adults, salivary secretion is generally used in the objective evaluation of oral dryness [49,50,51]. However, it is often impossible to evaluate salivation in older adults requiring long-term care because of deterioration in oral function [52,53]. In this study, clinical diagnostic criteria [26] were selected based on the participants’ characteristics. This evaluation method is a clinical diagnostic standard by visual inspection. The standard evaluation examines the amount of saliva and the dryness and wetness of the oral mucosa, and could be used for screening because it correlates with other methods, such as subjective symptoms, saliva wetness tester, and oral mucosa moisture-checking meter [54]. Since saliva wetness can be evaluated in patients whose saliva volume was difficult to measure, it has been widely used in previous studies targeting older adults requiring long-term care [15,55,56,57,58]. This evaluation method was selected because it was useful in daily clinical diagnosis and can be easily performed.

This study has several limitations. First, the study did not consider the cause of death; thus, deaths unrelated to illness or aging, such as accidents, may have been included. Second, the results are from a specific community, which possibly has unique characteristics. As such, it cannot be definitively concluded that the results can be generalized. Third, the present study did not consider social factors that may affect the lives of older adults requiring long-term care, such as family structure and the content of the care services provided [42,59,60]. Further studies will be required in the future to address these factors. Finally, evaluation of oral dryness based on visual inspection may have resulted in some variation among examiners, compared with other quantitative evaluation such as measurement devices, even if precalibration was performed.

This study showed that the evaluation of oral dryness might be used in screening for mortality risk in older adults requiring long-term care who are being treated in different settings. Older adults requiring long-term care may not attend regular dental check-ups, due to difficulties in traveling to dental facilities and limited resources for home-visit medical care [61,62], limiting opportunities to identify oral problems. Within this context, it is noteworthy that oral dryness, which is easy to observe and evaluate, is associated with the life prognosis of community-dwelling older adults requiring long-term care. It is necessary to address oral cavity issues in patient’s homes and at facilities and hospitals; however, it is also important to clarify community issues involving these various facilities and consider measures for providing community-based support. In the future, it will be necessary to conduct studies in other communities and to include oral dryness as one factor to be examined.

## 5. Conclusions

This study’s findings indicate that oral dryness is related to the life prognosis of older adults who require long-term care and receive that care in different settings. The results suggest that it is important to inform care workers and nurses about oral dryness, conduct regular oral function assessments, and identify and respond to mortality risks early.

## Figures and Tables

**Table 1:** Baseline characteristics comparison between survival and death groups.

	All (*n* = 289)		Survival (*n* = 187)		Death (*n* = 102)		*p*-Value
	*n* (%)	Median	*n* (%)	Median	*n* (%)	Median	
		(Q1,Q3)		(Q1,Q3)		(Q1,Q3)	
**Age (years)**		86		**85**		**88**	**<0.001 ^a^**
		(81,90)		(79,90)		(84,93)	
**Gender**							
Women	222(76.8)		**151(80.7)**		**71(69.6)**		**0.032 ^b^**
Men	67(23.2)		**36(19.3)**		**31(30.4)**		
**Observation period (days)**		1461		**1461**		**934**	**<0.001 ^a^**
		(1137,1461)		(1461,1461)		(632.5, 1197.8)	
Facility classification							
Home care	59(20.4)		42(22.5)		17(16.7)		0.115 ^b^
Elderly facility/medical ward	178(61.6)		107(57.2)		71(69.6)		
Group home for senile people	52(18.0)		38(20.3)		14(13.7)		
Medical history (presence)							
Respiratory disease	10(3.5)		5(2.7)		5(4.9)		0.322 ^b^
Aspiration pneumonia	7(2.4)		4(2.1)		3(2.9)		0.672 ^b^
Cerebrovascular disorder	105(36.3)		67(35.8)		38(37.3)		0.810 ^b^
Circulatory disorder	90(31.1)		56(29.9)		34(33.3)		0.552 ^b^
Neoplastic disease	21(7.3)		12(6.4)		9(8.8)		0.451 ^b^
Neurological disease	7(2.4)		5(2.7)		2(2.0)		0.706 ^b^
Parkinson’s disease	8(2.8)		6(3.2)		2(2.0)		0.537 ^b^
Diabetes mellitus	48(16.6)		27(14.4)		21(20.6)		0.179 ^b^
Number of medications							
≦3 types	66(22.8)		35(18.7)		31(30.4)		0.067 ^b^
≧4 types	213(73.7)		146(78.1)		67(65.7)		
Unknown	10(3.5)		6(3.2)		4(3.9)		
**BI (score)**		45		**55**		**15**	**<0.001 ^a^**
		(10,7)		(15,80)		(5,50)	
**CDR (score)**							
0/0.5	51(17.6)		**38(20.3)**		**13(12.7)**		**0.001 ^b^**
1	78(27.0)		**61(32.6)**		**17(16.7)**		
2	87(30.1)		**52(27.8)**		**35(34.3)**		
3	73(25.3)		**36(19.3)**		**37(36.3)**		
**BMI (kg/m^2^)**		21.3		**22.2**		**20.2**	**<0.001 ^a^**
		(19.1, 24.5)		(19.8, 25.3)		(17.7, 23.1)	
**MNA^®^-SF (score)**							
Normal nutritional status	83(28.8)		**66(35.3)**		**17(16.8)**		**<0.001 ^b^**
At risk of malnutrition	153(53.1)		**99(52.9)**		**54(53.5)**		
Malnourished	52(18.1)		**22(11.8)**		**30(29.7)**		
**Food type**							
Regular diet	136(47.1)		**101(54.0)**		**35(34.3)**		**0.001 ^b^**
Defined formula diet	153(52.9)		**86(46.0)**		**67(65.7)**		
Number of present teeth		0		0		0	0.236 ^a^
		(0,5)		(0,6)		(0, 4.3)	
**Rinsing ability (poor)**	76(26.3)		**37(19.8)**		**39(38.2)**		**0.001 ^b^**
**Articulation of/TA/(poor)**	60(20.8)		**31(16.6)**		**29(28.4)**		**0.018 ^b^**
**MWST (poor)**	58(20.1)		**30(16.0)**		**28(27.5)**		**0.021 ^b^**
State of dental plaque (present)	102(35.3)		64(34.2)		38(37.3)		0.606 ^b^
State of tongue coating (present)	151(52.2)		98(52.4)		53(52.0)		0.942 ^b^
**Oral dryness (present)**	57(19.7)		**30(16.0)**		**27(26.5)**		**0.033 ^b^**

BI, Barthel index; CDR, clinical dementia rating; BMI, body mass index; MNA^®^-SF, mini nutritional assessment-short form; MWST, modified water swallowing test; n, number; Q1, first quartile; Q3, third quartile; a, Mann–Whitney U test; b, χ^2^ test. Bold text indicates a statistically significant association (*p* < 0.05).

**Table 2:** Predictors of mortality according to Cox proportional-hazards regression.

Variable	H.R.	95%CI			*p*-Value
Age	1.07	1.03	−	1.11	<0.001
Gender (woman = 1)	0.38	0.23	−	0.63	<0.001
BI	0.98	0.97	−	1.00	0.006
Medical history (yes = 1)					
Aspiration pneumonia	1.33	0.36	−	5.01	0.670
Cerebrovascular disorder	0.97	0.63	−	1.51	0.901
Circulatory disorder	1.17	0.74	−	1.86	0.503
Neoplastic disease	1.98	0.94	−	4.17	0.071
**Diabetes mellitus**	**1.80**	**1.07**	**−**	**3.04**	**0.028**
Number of medications (≧3 types = 1)	0.91	0.56	−	1.46	0.682
CDR					
0/0.5	Reference				
1	0.74	0.33	−	1.66	0.471
2	0.99	0.46	−	2.14	0.983
3	1.31	0.53	−	3.23	0.559
MNA^®^-SF					
Normal nutritional status	Reference				
At risk of malnutrition	1.17	0.60	−	2.31	0.641
Malnourished	1.66	0.74	−	3.74	0.218
Food morphology (defined formula diet = 1)	0.86	0.47	−	1.56	0.621
Rinsing ability (poor = 1)	1.18	0.70	−	2.00	0.538
Articulation of /TA/ (poor = 1)	0.71	0.39	−	1.31	0.272
MWST (poor = 1)	0.92	0.54	−	1.57	0.770
**Oral dryness (yes = 1)**	**1.83**	**1.12**	**−**	**3.00**	**0.015**

HR, hazard ratio; CI, confidence interval; BI, Barthel index; CDR, clinical dementia rating; MNA^®^-SF, mini nutritional assessment-short form; MWST, modified water swallowing test. Bold text indicates a statistically significant association (*p* < 0.05).

## Data Availability

The data of the present study were used under license for the current study and, therefore, are not publicly available.
